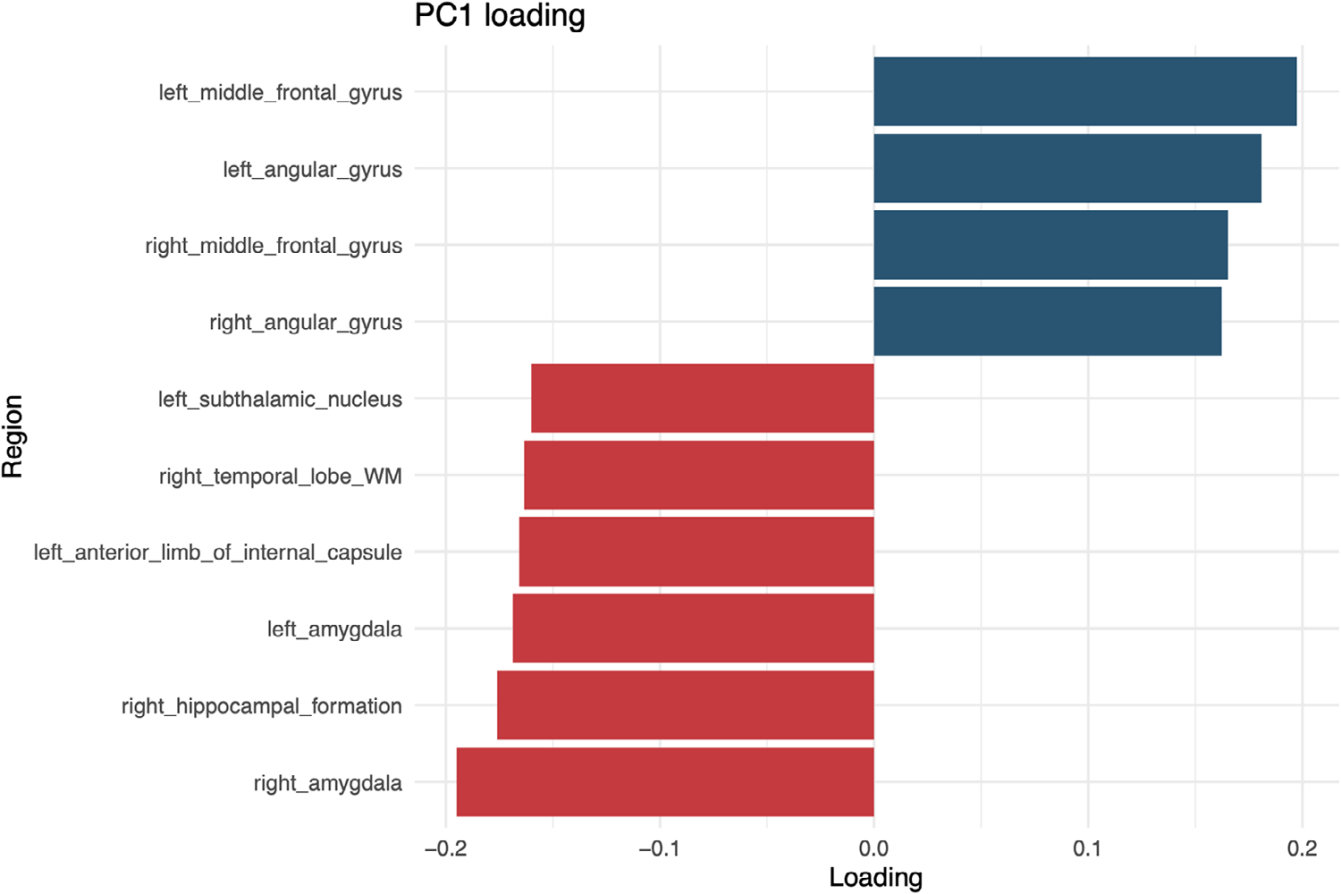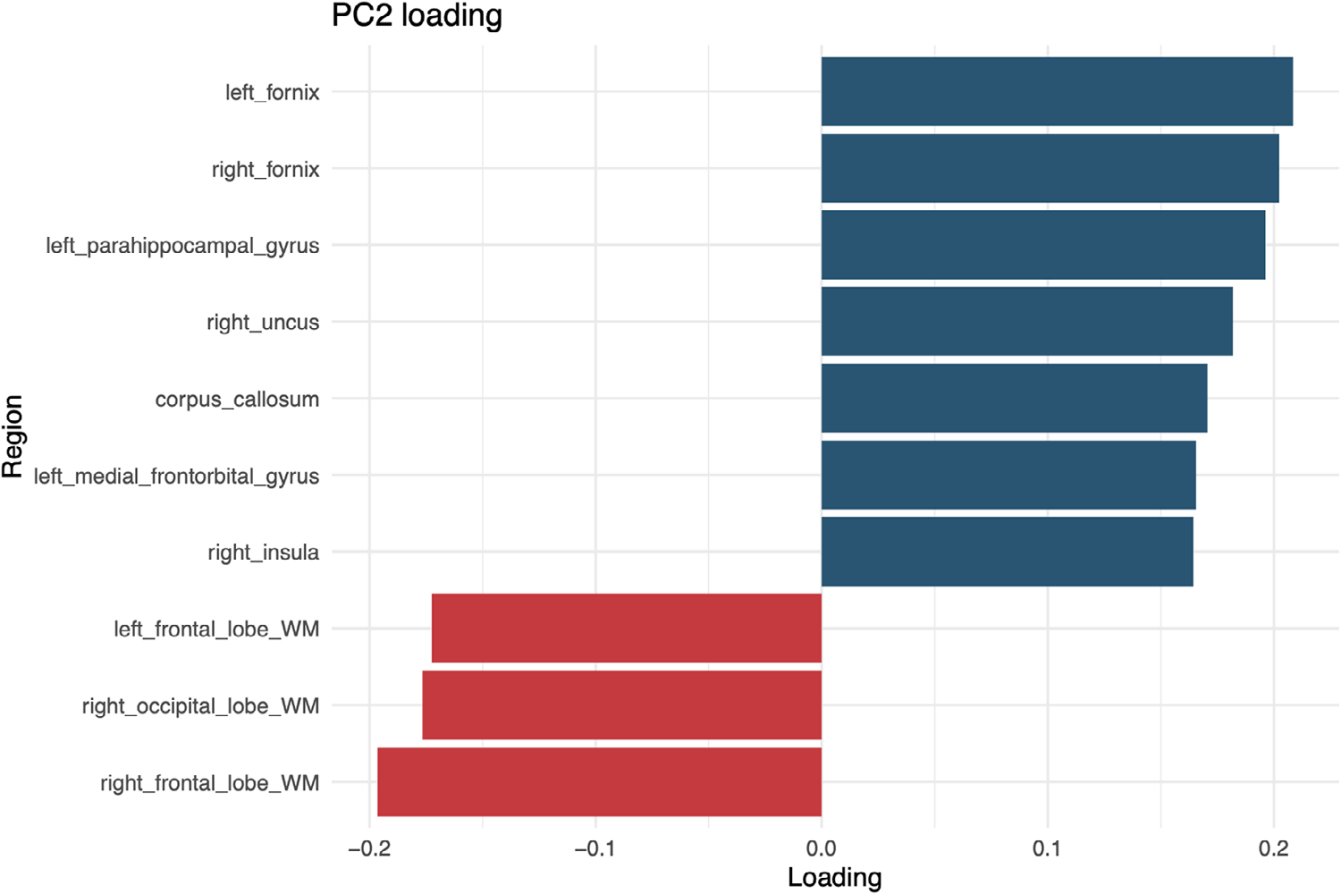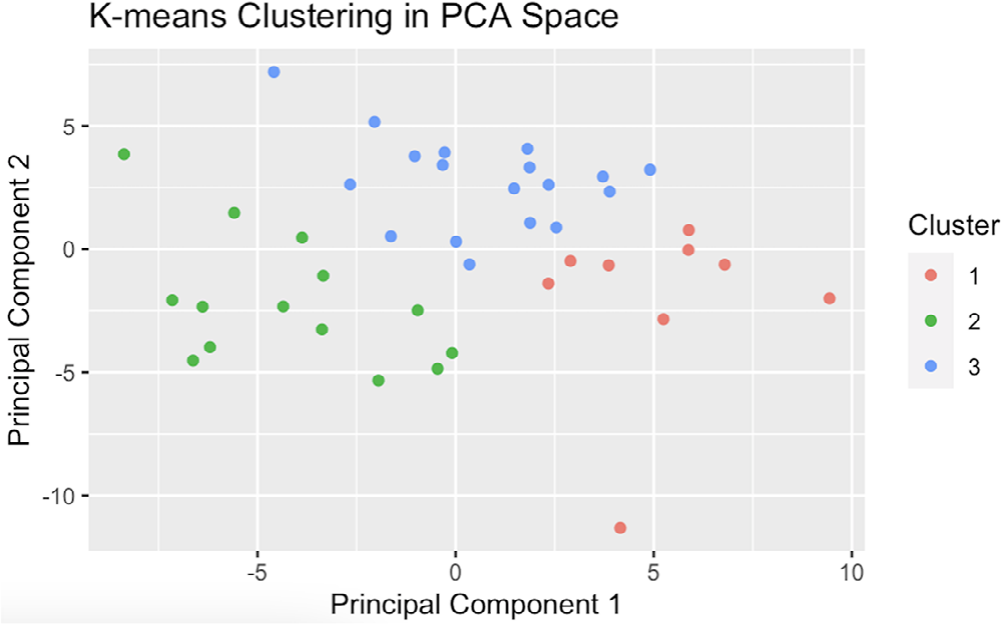# Is FDG‐PET metabolism associated with cognitive performance in long‐Covid patients?

**DOI:** 10.1002/alz.091297

**Published:** 2025-01-09

**Authors:** Micaela A Hernández, Luiza Santos Machado, Marco De Bastiani, Thomas Hugentobler Schlickmann, João Pedro Ferrari‐Souza, Agostina Carello, Belén Helou, Lucia Crivelli, Emilia Osa Sanz, Yanina Bérgamo, Silvia Vazquez, Gustavo Sevlever, Ricardo Allegri, Eduardo R. Zimmer, Ismael Luis Calandri

**Affiliations:** ^1^ Fleni, Buenos Aires, Buenos Aires Argentina; ^2^ Universidade Federal do Rio Grande do Sul, Porto Alegre, Rio Grande do Sul Brazil

## Abstract

**Background:**

Current evidence indicates that COVID‐19 infection can lead to neurological complications that persist beyond 12 weeks of infection (long‐COVID), often associated with cognitive decline. However, the underlying mechanisms remain unclear. Our aim is to study the brain metabolism of patients with long‐COVID and its association with cognitive performance.

**Method:**

Individuals with cognitive complaints for at least a month after COVID‐19 infection from an Argentine cohort of long‐COVID. Their brain glucose metabolism was assessed by FDG‐PET imaging, and the cognitive symptoms were monitored using cognitive evaluation. The FDG‐PET images were normalized using the individuals’ global mean value, and regions of interest mean signal was extracted using ICBM152 atlas. After applying Principal Components Analysis (PCA) to reduce dimensionality, we conducted clustering using K‐means with the two primary components extracted to create groups with similar metabolism patterns. With the neuropsychological data, we generated seven composites covering cognitive domains based on z‐scored data relative to the normal Argentine population. We conducted pairwise T‐tests to compare cognitive performance of metabolic clusters.

**Result:**

Forty‐one subjects were recruited, 27 were female. Mean age of 55 years (±12) with an average of 15 years of education (±2.3). In PC1, key contributing regions included the right medial temporal lobe, right hippocampus and bilateral amygdala, exhibiting hypometabolism, and bilateral frontal lobe, displaying preserved metabolism (Figure 1). PC2 was characterized by hypometabolism in bilateral frontal lobe and right occipital lobe (Figure 2). Three distinct clusters were identified: Cluster 1 and 2, differentiated by PC1 and Cluster 3, distinguished from Cluster 1 and 2 by PC2 (Figure 3). Regarding cognitive assessments, we observed statistically significant differences between Cluster 1 and 2 in executive composite (p=0.049) and global composite (p=0.025).

**Conclusion:**

Our study identified three distinct clusters based on brain metabolism, with differences in executive functions between the two of them. We consider that FDG‐PET only partially explains cognitive performance, mood and neural networks are probably relevant contributing factors to cognition.